# Publisher Correction: LifeTime and improving European healthcare through cell-based interceptive medicine

**DOI:** 10.1038/s41586-021-03287-8

**Published:** 2021-03-17

**Authors:** Nikolaus Rajewsky, Geneviève Almouzni, Stanislaw A. Gorski, Stein Aerts, Ido Amit, Michela G. Bertero, Christoph Bock, Annelien L. Bredenoord, Giacomo Cavalli, Susanna Chiocca, Hans Clevers, Bart De Strooper, Angelika Eggert, Jan Ellenberg, Xosé M. Fernández, Marek Figlerowicz, Susan M. Gasser, Norbert Hubner, Jørgen Kjems, Jürgen A. Knoblich, Grietje Krabbe, Peter Lichter, Sten Linnarsson, Jean-Christophe Marine, John C. Marioni, Marc A. Marti-Renom, Mihai G. Netea, Dörthe Nickel, Marcelo Nollmann, Halina R. Novak, Helen Parkinson, Stefano Piccolo, Inês Pinheiro, Ana Pombo, Christian Popp, Wolf Reik, Sergio Roman-Roman, Philip Rosenstiel, Joachim L. Schultze, Oliver Stegle, Amos Tanay, Giuseppe Testa, Dimitris Thanos, Fabian J. Theis, Maria-Elena Torres-Padilla, Alfonso Valencia, Céline Vallot, Alexander van Oudenaarden, Marie Vidal, Thierry Voet, LifeTime Community Working Groups, Lavinia Alberi, Stephanie Alexander, Theodore Alexandrov, Ernest Arenas, Claudia Bagni, Robert Balderas, Andrea Bandelli, Burkhard Becher, Matthias Becker, Niko Beerenwinkel, Monsef Benkirane, Marc Beyer, Wendy A. Bickmore, Erik E. A. L. Biessen, Niklas Blomberg, Ingmar Blumcke, Bernd Bodenmiller, Barbara Borroni, Dimitrios T. Boumpas, Thomas Bourgeron, Sarion Bowers, Dries Braeken, Catherine Brooksbank, Nils Brose, Hilgo Bruining, Jo Bury, Nicolo Caporale, Giorgio Cattoretti, Nadia Chabane, Hervé Chneiweiss, Stuart A. Cook, Paolo Curatolo, Marien I. de Jonge, Bart Deplancke, Bart De Strooper, Peter de Witte, Stefanie Dimmeler, Bogdan Draganski, Anna Drews, Costica Dumbrava, Stefan Engelhardt, Thomas Gasser, Evangelos J. Giamarellos-Bourboulis, Caroline Graff, Dominic Grün, Ivo G. Gut, Oskar Hansson, David C. Henshall, Anna Herland, Peter Heutink, Stephane R. B. Heymans, Holger Heyn, Meritxell Huch, Inge Huitinga, Paulina Jackowiak, Karin R. Jongsma, Laurent Journot, Jan Philipp Junker, Shauna Katz, Jeanne Kehren, Stefan Kempa, Paulus Kirchhof, Christine Klein, Natalia Koralewska, Jan O. Korbel, Malte Kühnemund, Angus I. Lamond, Elsa Lauwers, Isabelle Le Ber, Ville Leinonen, Alejandro López-Tobón, Emma Lundberg, Astrid Lunkes, Henrike Maatz, Matthias Mann, Luca Marelli, Vera Matser, Paul M. Matthews, Fatima Mechta-Grigoriou, Radhika Menon, Anne F. Nielsen, Massimiliano Pagani, R. Jeroen Pasterkamp, Asla Pitkänen, Valentin Popescu, Cyril Pottier, Alain Puisieux, Rosa Rademakers, Dory Reiling, Orly Reiner, Daniel Remondini, Craig Ritchie, Jonathan D. Rohrer, Antoine-Emmanuel Saliba, Raquel Sanchez-Valle, Amedeo Santosuosso, Arnold Sauter, Richard A. Scheltema, Philip Scheltens, Herbert B. Schiller, Anja Schneider, Philip Seibler, Kelly Sheehan-Rooney, David J. Shields, Kristel Sleegers, August B. Smit, Kenneth G. C. Smith, Ilse Smolders, Matthis Synofzik, Wai Long Tam, Sarah A. Teichmann, Maria Thom, Margherita Y. Turco, Heleen M. M. van Beusekom, Rik Vandenberghe, Silvie Van den Hoecke, Ibo van de Poel, Andre van der Ven, Julie van der Zee, Jan van Lunzen, Geert van Minnebruggen, Alexander van Oudenaarden, Wim Van Paesschen, John C. van Swieten, Remko van Vught, Matthijs Verhage, Patrik Verstreken, Carlo Emanuele Villa, Jörg Vogel, Christof von Kalle, Jörn Walter, Sarah Weckhuysen, Wilko Weichert, Louisa Wood, Anette-Gabriele Ziegler, Frauke Zipp

**Affiliations:** 1 Berlin Institute for Medical Systems Biology, Max Delbrück Center for Molecular Medicine in the Helmholtz Association, Berlin, Germany; 2 Charité-Universitätsmedizin, Berlin, Germany; 3 Berlin Institute of Health (BIH), Berlin, Germany; 4 German Center for Cardiovascular Research (DZHK), Partner Site Berlin, Berlin, Germany; 5 Institut Curie, CNRS, PSL Research University, Sorbonne Université, Nuclear Dynamics Unit, Equipe Labellisée Ligue contre le cancer, Paris, France; 6 VIB Center for Brain and Disease Research, Leuven, Belgium; 7 Department of Human Genetics, KU Leuven, Leuven, Belgium; 8 Department of Immunology, Weizmann Institute of Science, Rehovot, Israel; 9 Centre for Genomic Regulation (CRG), Barcelona Institute of Science and Technology, Barcelona, Spain; 10 CeMM Research Center for Molecular Medicine of the Austrian Academy of Sciences, Vienna, Austria; 11 Department of Laboratory Medicine, Medical University of Vienna, Vienna, Austria; 12 Ludwig Boltzmann Institute for Rare and Undiagnosed Diseases, Vienna, Austria; 13 Department of Medical Humanities, Julius Center for Health Sciences and Primary Care, University Medical Center Utrecht, Utrecht, The Netherlands; 14 Institute of Human Genetics, UMR 9002, CNRS and University of Montpellier, Montpellier, France; 15 Department of Experimental Oncology, IEO, European Institute of Oncology IRCCS, Milan, Italy; 16 Hubrecht Institute, Royal Netherlands Academy of Arts and Sciences (KNAW), Utrecht, The Netherlands; 17 University Medical Center Utrecht, Utrecht, The Netherlands; 18 Oncode Institute, Utrecht, The Netherlands; 19 The Princess Máxima Center for Pediatric Oncology, Utrecht, The Netherlands; 20 Department of Neurosciences, KU Leuven, Leuven, Belgium; 21 UK Dementia Research Institute at UCL, University College London, London, UK; 22 Department of Pediatric Oncology/Hematology, Charité-Universitätsmedizin Berlin, Berlin, Germany; 23 Cell Biology and Biophysics Unit, European Molecular Biology Laboratory, Heidelberg, Germany; 24 Institut Curie, PSL Research University, Paris, France; 25 Institute of Bioorganic Chemistry, Polish Academy of Sciences, Poznan, Poland; 26 Institute of Computing Science, Poznan University of Technology, Poznan, Poland; 27 Friedrich Miescher Institute for Biomedical Research, Basel, Switzerland; 28 Faculty of Natural Sciences, University of Basel, Basel, Switzerland; 29 Cardiovascular and Metabolic Sciences, Max Delbrück Center for Molecular Medicine in the Helmholtz Association (MDC), Berlin, Germany; 30 Department of Molecular Biology and Genetics (MBG), Aarhus University, Aarhus, Denmark; 31 Interdisciplinary Nanoscience Centre (iNANO), Aarhus University, Aarhus, Denmark; 32 Institute of Molecular Biotechnology of the Austrian Academy of Sciences (IMBA), Vienna, Austria; 33 Medical University of Vienna, Vienna, Austria; 34 Division of Molecular Genetics, German Cancer Research Center (DKFZ), Heidelberg, Germany; 35 Division of Molecular Neurobiology, Department of Medical Biochemistry and Biophysics, Karolinska Institutet, Stockholm, Sweden; 36 Science for Life Laboratory, Stockholm, Sweden; 37 Laboratory for Molecular Cancer Biology, VIB Center for Cancer Biology, KU Leuven, Leuven, Belgium; 38 Department of Oncology, KU Leuven, Leuven, Belgium; 39 European Molecular Biology Laboratory, European Bioinformatics Institute, Wellcome Genome Campus, Cambridge, UK; 40 Cancer Research UK Cambridge Institute, University of Cambridge, Cambridge, UK; 41 Wellcome Sanger Institute, Wellcome Genome Campus, Cambridge, UK; 42 CNAG-CRG, Centre for Genomic Regulation, Barcelona Institute of Science and Technology, Barcelona, Spain; 43 Universitat Pompeu Fabra, Barcelona, Spain; 44 ICREA, Barcelona, Spain; 45 Department of Internal Medicine, Radboud Center for Infectious Diseases, Radboud University Medical Center, Nijmegen, The Netherlands; 46 Radboud Institute for Molecular Life Sciences, Radboud University Medical Center, Nijmegen, The Netherlands; 47 Life and Medical Sciences Institute (LIMES), University of Bonn, Bonn, Germany; 48 Centre de Biochimie Structurale, CNRS UMR 5048, INSERM U1054, Université de Montpellier, Montpellier, France; 49 VIB Technology Watch, Ghent, Belgium; 50 Department of Molecular Medicine, University of Padua School of Medicine, Padua, Italy; 51 IFOM, The FIRC Institute of Molecular Oncology, Padua, Italy; 52 Institute for Biology, Humboldt University of Berlin, Berlin, Germany; 53 Epigenetics Programme, Babraham Institute, Cambridge, UK; 54 Centre for Trophoblast Research, University of Cambridge, Cambridge, UK; 55 Department of Translational Research, Institut Curie, PSL Research University, Paris, France; 56 Institute of Clinical Molecular Biology, Kiel University, Kiel, Germany; 57 University Hospital Schleswig-Holstein, Campus Kiel, Kiel, Germany; 58 German Center for Neurodegenerative Diseases (DZNE), Bonn, Germany; 59 PRECISE, Platform for Single Cell Genomics and Epigenomics at the German Center for Neurodegenerative Diseases and the University of Bonn, Bonn, Germany; 60 Division of Computational Genomics and Systems Genetics, German Cancer Research Center (DKFZ), Heidelberg, Germany; 61 Genome Biology Unit, European Molecular Biology Laboratory, Heidelberg, Germany; 62 Department of Computer Science and Applied Mathematics, Weizmann Institute of Science, Rehovot, Israel; 63 Department of Oncology and Hemato-oncology, University of Milan, Milan, Italy; 64 Human Technopole, Milan, Italy; 65 Biomedical Research Foundation, Academy of Athens, Athens, Greece; 66 Institute of Computational Biology, Helmholtz Zentrum München - German Research Center for Environmental Health, Neuherberg, Germany; 67 Department of Mathematics, Technical University of Munich, Munich, Germany; 68 Institute of Epigenetics and Stem Cells (IES), Helmholtz Zentrum München - German Research Center for Environmental Health, Munich, Germany; 69 Faculty of Biology, Ludwig-Maximilians Universität, Munich, Germany; 70 Barcelona Supercomputing Center (BSC), Barcelona, Spain; 71 CNRS UMR3244, Institut Curie, PSL University, Paris, France; 72 Department of Medicine, University of Fribourg, Fribourg, Switzerland; 73 Swiss Integrative Center for Human Health SA (SICHH), Fribourg, Switzerland; 74 Structural and Computational Biology Unit, European Molecular Biology Laboratory, Heidelberg, Germany; 75 Skaggs School of Pharmacy and Pharmaceutical Sciences, University of California San Diego, La Jolla, CA USA; 76 Department of Medical Biochemistry and Biophysics, Karolinska Institutet, Stockholm, Sweden; 77 Department of Fundamental Neurosciences, University of Lausanne, Lausanne, Switzerland; 78 Department of Biomedicine and Prevention, University of Rome Tor Vergata, Rome, Italy; 79 Becton Dickinson, San Jose, CA USA; 80 Science Gallery International, Dublin, Ireland; 81 Unit of Inflammation Research, Institute of Experimental Immunology, University of Zurich, Zurich, Switzerland; 82 Department of Biosystems Science and Engineering, ETH Zurich, Basel, Switzerland; 83 Swiss Institute of Bioinformatics, Lausanne, Switzerland; 84 Institut de Génétique Humaine, Université de Montpellier, Laboratoire de Virologie Moléculaire CNRS-UMR9002, Montpellier, France; 85 MRC Human Genetics Unit, Institute of Genetics and Molecular Medicine, University of Edinburgh, Edinburgh, UK; 86 Department of Pathology, Cardiovascular Research Institute Maastricht, Maastricht University, Maastricht, The Netherlands; 87 Institute for Molecular Cardiovascular Research, RWTH University Hospital Aachen, Aachen, Germany; 88 ELIXIR Hub, Wellcome Genome Campus, Cambridge, UK; 89 Neuropathologisches Institut, Universikätsklinikum, Erlangen, Germany; 90 Department of Quantitative Biomedicine, University of Zurich, Zurich, Switzerland; 91 Department of Clinical and Experimental Sciences, University of Brescia, Brescia, Italy; 92 Fourth Department of Internal Medicine, School of Medicine, National & Kapodistrian University of Athens, Athens, Greece; 93 University of Cyprus Medical School, Nicosia, Cyprus; 94 Human Genetics and Cognitive Functions Unit, Institut Pasteur, UMR 3571, CNRS, Université de Paris, Paris, France; 95 Imec, Leuven, Belgium; 96 Department of Molecular Neurobiology, Max Planck Institute of Experimental Medicine, Göttingen, Germany; 97 Department of Child and Adolescent Psychiatry, Amsterdam UMC, Amsterdam, The Netherlands; 98 Flanders Institute for Biotechnology (VIB), Ghent, Belgium; 99 Department of Medicine & Surgery, Università degli studi di Milano-Bicocca, Milan, Italy; 100 Centre cantonal autisme, Département de psychiatrie, CHUV, Allières, Lausanne, Switzerland; 101 Institut National de la Santé et de la Recherche Medicale (INSERM), Paris, France; 102 Sorbonne Universités, Paris, France; 103 Centre National de la Recherche Scientifique (CNRS), Paris, France; 104 National Heart and Lung Institute, Imperial College London, London, UK; 105 MRC-London Institute of Medical Sciences, Hammersmith Hospital Campus, London, UK; 106 Program in Cardiovascular and Metabolic Disorders, Duke-National University of Singapore, Singapore, Singapore; 107 National Heart Research Institute Singapore (NHRIS), National Heart Centre Singapore, Singapore, Singapore; 108 Department of System Medicine, University of Rome Tor Vergata, Rome, Italy; 109 Department of Laboratory Medicine, Radboud Center for Infectious Diseases, Radboud University Medical Center, Nijmegen, The Netherlands; 110 Institute of Bioengineering, Ecole Polytechnique Fédérale de Lausanne (EPFL), Lausanne, Switzerland; 111 Department of Pharmaceutical and Pharmacological Sciences, University of Leuven, Leuven, Belgium; 112 Institute for Cardiovascular Regeneration, Goethe University, Frankfurt, Germany; 113 Department of Clinical Neurosciences, Lausanne University Hospital and University of Lausanne, Lausanne, Switzerland; 114 Department of Neurology, Max-Planck Institute for Human Cognitive and Brain Sciences, Leipzig, Germany; 115 Communication Networks, Content & Technology, European Commission, Brussels, Belgium; 116 Institute of Pharmacology and Toxicology, Technische Universität München, Munich, Germany; 117 Department for Neurodegenerative Diseases, Hertie Institute for Clinical Brain Research, University of Tübingen, Tübingen, Germany; 118 German Center for Neurodegenerative Diseases, Tübingen, Germany; 119 Hellenic Institute for the Study of Sepsis, Athens, Greece; 120 Department of NVS, Division of Neurogeriatrics, Karolinska Institutet, Stockholm, Sweden; 121 Unit of Hereditary Dementia, Karolinska University Hospital-Solna, Stockholm, Sweden; 122 Max-Planck-Institute of Immunobiology and Epigenetics, Freiburg, Germany; 123 Centre for Integrative Biological Signaling Studies, University of Freiburg, Freiburg, Germany; 124 Clinical Memory Research Unit, Lund University, Lund, Sweden; 125 Memory Clinic, Skåne University Hospital, Malmö, Sweden; 126 FutureNeuro SFI Research Centre, Royal College of Surgeons in Ireland, Dublin, Ireland; 127 Division of Micro- and Nanosystems, KTH Royal Institute of Technology, Stockholm, Sweden; 128 Hertie Institute for Clinical Brain Research, Tübingen, Germany; 129 Department of Cardiology, Cardiovascular Research Institute Maastricht (CARIM), Maastricht University Medical Centre, Maastricht, The Netherlands; 130 Department of Cardiovascular Research, University of Leuven, Leuven, Belgium; 131 Netherlands Heart Institute (ICIN), Utrecht, The Netherlands; 132 Max Planck Institute of Molecular Cell Biology and Genetics, Dresden, Germany; 133 Swammerdam Institute for Life Sciences, University of Amsterdam, Amsterdam, The Netherlands; 134 Netherlands Institute for Neuroscience, Amsterdam, The Netherlands; 135 Montpellier GenomiX (MGX), Institut de Génomique Fonctionnelle, Montpellier, France; 136 Bayer AG Pharmaceuticals, Berlin, Germany; 137 Institute of Cardiovascular Sciences, University of Birmingham, Birmingham, UK; 138 Department of Cardiology, University Heart and Vascular Center Hamburg, Hamburg, Germany; 139 Sandwell and West Birmingham and University Hospitals Birmingham NHS Trusts, Birmingham, UK; 140 German Center for Cardiovascular Research (DZHK), Partner Site Hamburg/Kiel/Lübeck, Hamburg, Germany; 141 Institute of Neurogenetics, University of Lübeck, Lübeck, Germany; 142 CARTANA, Stockholm, Sweden; 143 Centre for Gene Regulation and Expression, University of Dundee, Dundee, UK; 144 Department of Neurology, Hôpital La Pitié Salpêtrière, Paris, France; 145 Neurocenter, Neurosurgery, Kuopio University Hospital and Institute of Clinical Medicine, University of Eastern Finland, Kuopio, Finland; 146 Unit of Clinical Neuroscience, Neurosurgery, University of Oulu and Medical Research Center Oulu, Oulu University Hospital, Oulu, Finland; 147 Science for Life Laboratory, KTH - Royal Institute of Technology, Stockholm, Sweden; 148 Department of Proteomics and Signal Transduction, Max Planck Institute of Biochemistry, Martinsried, Germany; 149 Proteomics Program, Novo Nordisk Foundation Center for Protein Research, University of Copenhagen, Copenhagen, Denmark; 150 Centre for Sociological Research, KU Leuven, Leuven, Belgium; 151 Department of Medical Biotechnology and Translational Medicine, University of Milan, Milan, Italy; 152 Department of Brain Sciences, Imperial College London, London, UK; 153 UK Dementia Research Institute at Imperial College London, London, UK; 154 Institut Curie, Stress and Cancer Laboratory, Equipe labélisée par la Ligue Nationale contre le Cancer, PSL Research University, Paris, France; 155 MIMETAS, Leiden, The Netherlands; 156 IFOM, The FIRC Institute of Molecular Oncology, Milan, Italy; 157 Department of Translational Neuroscience, UMC Utrecht Brain Center, University Medical Center Utrecht, Utrecht University, Utrecht, The Netherlands; 158 A. I. Virtanen Institute for Molecular Sciences, University of Eastern Finland, Kuopio, Finland; 159 VIB Center for Molecular Neurology, Antwerp, Belgium; 160 Department of Biomedical Sciences, University of Antwerp, Antwerp, Belgium; 161 District Court, Amsterdam, The Netherlands and Court of Appeal, The Hague, The Netherlands; 162 Department of Molecular Genetics, Weizmann Institute of Science, Rehovot, Israel; 163 Department of Physics and Astronomy, Bologna University, Bologna, Italy; 164 Centre for Clinical Brain Sciences, University of Edinburgh, Edinburgh, Scotland UK; 165 Dementia Research Centre, Department of Neurodegenerative Disease, UCL Queen Square Institute of Neurology, University College London, London, UK; 166 Helmholtz Institute for RNA-based Infection Research (HIRI), Helmholtz-Center for Infection Research (HZI), Würzburg, Germany; 167 Alzheimer’s Disease and Other Cognitive Disorders Unit, Fundació Clínic per a la Recerca Biomèdica, Institut d’Investigacions Biomèdiques August Pi i Sunyer (IDIBAPS), Universitat de Barcelona, Barcelona, Spain; 168 European Center for Law, Science and new Technologies (ECLT), University of Pavia, Pavia, Italy; 169 Department of Law, University of Pavia, Pavia, Italy; 170 Institute of Advanced Studies (IUSS), Pavia, Italy; 171 World Commission on the Ethics of Scientific Knowledge and Technology (COMEST-UNESCO), Paris, France; 172 Office of Technology Assessment at the German Parliament, Berlin, Germany; 173 Biomolecular Mass Spectrometry and Proteomics, Bijvoet Center for Biomolecular Research and Utrecht Institute for Pharmaceutical Sciences, University of Utrecht, Utrecht, The Netherlands; 174 Netherlands Proteomics Center, Utrecht, The Netherlands; 175 Alzheimer Center, Amsterdam University Medical Center, Amsterdam, The Netherlands; 176 Institute of Lung Biology and Disease, German Center for Lung Research (DZL), Helmholtz Zentrum München, Munich, Germany; 177 Department of Neurodegenerative Diseases and Geriatric Psychiatry, University Bonn, Bonn, Germany; 178 Oncology R&D, Pfizer Inc, San Diego, CA USA; 179 Department of Molecular and Cellular Neurobiology, Center for Neurogenomics and Cognitive Research, Amsterdam Neuroscience, VU University Amsterdam, Amsterdam, The Netherlands; 180 Department of Medicine, University of Cambridge, Cambridge, UK; 181 Cambridge Institute of Therapeutic Immunology and Infectious Disease, Jeffrey Cheah Biomedical Centre, University of Cambridge, Cambridge, UK; 182 Department of Pharmaceutical Sciences, Center for Neurosciences (C4N), Vrije Universiteit Brussel, Brussels, Belgium; 183 Department of Physics, Cavendish Laboratory, Cambridge, UK; 184 Division of Neuropathology, National Hospital for Neurology and Neurosurgery, London, UK; 185 Department of Clinical and Experimental Epilepsy, UCL Queen Square Institute of Neurology, London, UK; 186 Department of Pathology, University of Cambridge, Cambridge, UK; 187 Department of Cardiology, Erasmus MC University Medical Center, Rotterdam, The Netherlands; 188 Department of Neurology, University Hospital Leuven, KU Leuven, Leuven, Belgium; 189 Department of Values, Technology and Innovation, Delft University of Technology, Delft, The Netherlands; 190 ViiV Healthcare, London, UK; 191 University Medical Center, Hamburg, Germany; 192 Department of Neurosciences, University Hospital Leuven, KU Leuven, Leuven, Belgium; 193 Department of Neurology, Erasmus Medical Centre, University Medical Center Rotterdam, Rotterdam, The Netherlands; 194 Department of Functional Genomics, Center for Neurogenomics and Cognitive Research, Vrije Universiteit Amsterdam, Amsterdam, The Netherlands; 195 Department of Clinical Genetics, Center for Neurogenomics and Cognitive Research, Amsterdam University Medical Center, Amsterdam, The Netherlands; 196 Institute of Molecular Infection Biology, University of Würzburg, Würzburg, Germany; 197 Department of Genetics, Saarland University, Saarbrücken, Germany; 198 Division of Neurology, Antwerp University Hospital, Antwerp, Belgium; 199 Institute of Pathology, Technical University Munich, Munich, Germany; 200 Babraham Institute, Babraham Research Campus, Cambridge, UK; 201 Institute of Diabetes Research, Helmholtz Zentrum München, Munich, Germany; 202 Technical University Munich, at Klinikum rechts der Isar, Munich, Germany; 203 Department of Neurology, University Medical Center of the Johannes Gutenberg University Mainz, Mainz, Germany

**Keywords:** Systems biology, Biomarkers, Diseases

Correction to: *Nature* 10.1038/s41586-020-2715-9Published online 07 September 2020

In this Perspective, owing to an error in the HTML, the surname of author Alejandro López-Tobón of the LifeTime Community Working Groups consortium was indexed as ‘Tobon’ rather than ‘López-Tobón’ and the accents were missing. The HTML version of the original Perspective has been corrected; the PDF and print versions were always correct.

